# Synthesis and characterization of hexagonal boron nitride used for comparison of removal of anionic and cationic hazardous azo-dye: kinetics and equilibrium studies

**DOI:** 10.3906/kim-2004-23

**Published:** 2020-12-16

**Authors:** Tuba TARHAN

**Affiliations:** 1 Vocational High School of Health Services, Mardin Artuklu University, Mardin Turkey

**Keywords:** Hexagonal boron nitride, nanostructure, Victoria blue B, Metanil yellow, comparison adsorption

## Abstract

The purpose of this study was to compare the adsorption behavior of cationic and anionic dyes onto a hexagonal boron nitride (hBN) nanostructure that was rich in a negative charge. Herein, the hBN nanostructure was synthesized using boric acid as a precursor material. The characteristic peaks of the hBN nanostructure were performed using Fourier transform infrared (FT-IR) and Raman spectroscopies. The morphology and the particle size of hBN nanostructure were determined by transmission electron microscopy (TEM) and scanning electron microscopy (SEM). During the studies, various essential adsorption parameters were investigated, such as the initial dye concentration, pH of the dye solution, adsorbent dose, and contact time. Under optimal conditions, the removal of 42.6% Metanil yellow (MY) and 90% Victoria blue B (VBB) from aqueous solution was performed using a 10-mg hBN nanostructure. Furthermore, the equilibrium studies showed that the Freundlich isotherm model fitted well for the removal of MY. However, the Langmuir isotherm model fitted well for the removal of VBB. Moreover, according to the results obtained from the kinetic studies, while the first-order kinetic model was suited for the adsorption of the MY, the second-order kinetic model was found to well fit for the adsorption of VBB.

## 1. Introduction

In the modern age, unnecessary industrial and anthropological activities cause numerous problems related to the environment. Moreover, these unneeded activities affect flora and fauna cause pollution of the water. Nowadays, countless problems related to the environment, such as industrial pollution, rapid population growth, production of toxic materials, and environmental pollutants, can be listed [1,2]. Despite the fact that researchers are working very hard on these environmental problems, the problems of water, air, and soil pollution remain an issue [3–5].

Generally, these environmental problems arise due to the largescale production of synthetic and organic materials [6]. Furthermore, these pollutants are widely used in leather, textile, shoe polish, dyeing and printing, colored water-fast inks, paper, cosmetic, and pharmaceutical industries. Dyes, pharmaceuticals, and other water pollutants are not easily decomposed in nature. Even low concentrations of dyes, pharmaceuticals, and their derivative products cause extremely toxic effects on aquatic life [7]. There are more than 100,000 commercial types of textile dyes and over 70 × 105 tons of the most dangerous chemical pollutants to the environment are produced annually[8]. The unprocessed industry effluents generally contain a largescale of dyes that cause many environmental problems, such as cytotoxicity [9], genotoxicity, reduce light penetration, and produce carcinogenic aromatic amines [10] in the aquatic environment [11,12]. Therefore, there is much research being conducted in this area.

Cationic dyes are important types of dyes because they are used the staining of microorganisms [13]. Moreover, Victoria blue B (VBB) (cationic dye) is a photosensitizer, which induces a cytotoxic response in several mammalian cell lines [14].

Metanil yellow (MY) is one of the best water-soluble anionic azo dyes. It is commonly used for industrial applications, such as dyeing leather, spirit lacquer, shoe polish, staining paper, colored water-fast inks, manufacturer of pigment lakes, etc. [15–17]. Although not allowed, it is commonly used as a colorant agent in many food industries. It causes numerous problems in health and the environment during processing and transforming. Therefore, MY is a major pollutant for water and aquatic life [15–18].

Over recent years, several adsorbents have been researched for the removal of pollutants, such as heavy metal ions, hazardous organic dyes, pharmaceuticals, and oils pollution from aqueous solutions. Usually, many adsorbents, such as active carbon, magnetic nanocomposite, graphene, hexagonal boron nitride (h-BN), zeolite, montmorillonite, carbonaceous nanofiber adsorbents, and mesoporous aluminum oxide, have been tested for the removal of dyes [19–22]. Hexagonal boron nitride (hBN) possesses preeminent physical and chemical properties, such as high chemical stability, temperature stability [23], low density, high thermal conductivity, good mechanical strength [24], antioxidation ability [25,26], and is environmentally friendly. Moreover, hBN possesses an outstanding adsorption rate and capacity for the removal of organic dyes in aqueous solution because of the combination of the 3-dimensional BN structure and rich adsorbing sites [27–34].

In this study, a hBN nanostructure was successfully synthesized from boric acid using a furnace and characterized by different analytical devices, and afterwards, it was used for comparison of the removal of anionic and cationic dye in aqueous solutions as an adsorbent.

## 2. Materials and methods

### 2.1. Materials

Boric acid was obtained from Diva Chemicals Agency Ltd. STI (Baoding, Hebei, China). Ammonium hydroxide was purchased from BDH Chemicals (Mir qap-kuwait City, Kuwait). Metanilyellow (MY) (C _18_ H _14_ N _3_ NaO _3_ S) and Victoria blue B (VBB) (C _33_ H _32_ ClN _3_ ) were obtained from Fluka (Fluka Chemie GmbH, Buchs, Switzerland). All of the reagents were used without further puriﬁcation.

### 2.2. Methods

#### 2.2.1. Synthesis of hBN

First, 2 g boric acid was weighed and dispersed in 3 mL of ammonia solution (13.38 M). The mixture was overlaid on a silicium carbide boat and heated on a hot plate at 100 °C for 20 min. Afterwards, the plate was placed in a furnace (Protherm PTF 14/50/450, Protherm Furnaces, Ankara, Turkey) and heated to 1300 °C (8 °C/min) under ammonia gas for approximately 3 h, and then retained at 1300 °C for 2 h. The product was taken out of the furnace at around 550 °C and scraped off onto the plate using a spatula [35].

#### 2.2.2. Dye adsorption procedure

First, 100 mL of dye solution and 10 mg of adsorbent (hBN) were placed into a 100-mL glass-stoppered flask at 25 °C and stirred at 200 rpm using a shaker for 24 h. While the experiments with the MY dye solution were conducted with concentrations of 7 mgL ^-1^ , 10 mgL ^-1^ , and 12 mgL ^-1^ , the experiments with the VBB dye solution were conducted with concentrations of 12 mgL ^-1^ , 15 mgL ^-1^ , and 20 mgL ^-1^ . In addition, each concentration value was studied time-dependent. The adsorbent was taken out of the solution at the end of each period by centrifugation at a speed of 15,000 rpm min–1 for 10 min. The absorbance of the supernatant solution in the equilibrium was measured using a UV/Vis spectrophotometer at 434 and 618 nm for the MY and VBB dyes, respectively [18,36]. In addition, the effect of pH, adsorbent dose, concentration of the dye solution, and adsorption time on the % removal of the anionic (MY) and cationic (VBB) dyes were studied. All of the experiments were tested in triplicate. The adsorption capacity at time t, *q* *t* (mgg ^-1^ ), was calculated using the Eq. (1):

(1)qt=(C0-Ct)VM

where *C*_*o*_ is the initial dye concentration of a solution (mgL ^-1^ ), *C*_*t*_ is the final concentration of dye solution at time t (mgL ^-1^ ), *M* is the weight of the adsorbent (g), and *V* is the volume of the dye solution (L).

The % removal of dye in the solution was determined using Eq. (2):

(2)Removal(%)=Ci-CfCix100

where *C*_*i*_ and *C*_*f*_ are the initial and final dye concentrations of a solution, before and after adsorption, respectively.

#### 2.2.3. Instruments

The synthesized hBN nanostructure was characterized using different analytical devices. The structure of the molecule was determined by Fourier transform infrared spectrometry (FTIR, Thermo NICOLET IS50 spectrometer; Thermo Fisher Scientific Inc., Waltham, MA, USA) and Raman spectrometry (Renishaw inVia reflex; Renishaw plc, Gloucestershire, UK). Raman spectroscopy measurement was performed using a Renishaw inVia reflex Raman spectrometer with a diode laser at 830 nm that was arranged to a 10-s exposure time and 50× objective (numerical aperture, 0.75) with a laser power of 50 mW. The particle size and morphology were characterized by scanning electron microscopy (SEM, Carl Zeiss EVO 40; Carl Zeiss Microscopy GmbH, Oberkochen, Germany), and transmission electron microscope (TEM, FEI TALOS F200S 200 kV; Thermo Fisher Scientific Inc.). Furthermore, some characteristic peaks of the dyes were determined by FTIR after adsorption of the dye molecules onto the hBN nanostructure. The equilibrium concentrations of the dye solutionswere measured at the maximum absorbance values using a UV/Vis spectrophotometer (PerkinElmer Lamda 25; PerkinElmer, Inc., Waltham, MA, USA).

## 3. Results and discussion

### 3.1. Characterizations

**Figure 1:**
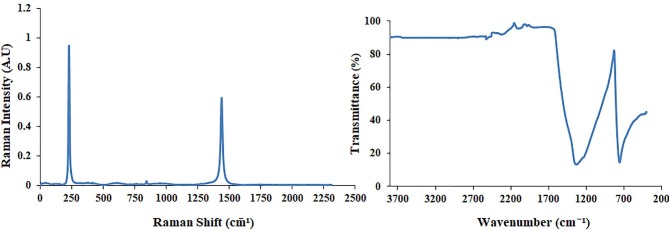
Raman (a) and FTIR (b) spectra of the pristine hBN nanostructure.

Figure 1a shows the Raman spectrum of the hBN nanostructure, where it can be seen that the major bands at around 322 and 1365 cm ^-1^ were attributed to CaF2 and hBNs, respectively [35]. Figure 1b shows the FTIR spectrum of the pristine hBN. The characteristic peaks of the B-N vibration were obtained at around 1371 and 821 cm ^-1^ , and showed that the hBN nanostructure was successfully synthesized [35].

**Figure 2:**
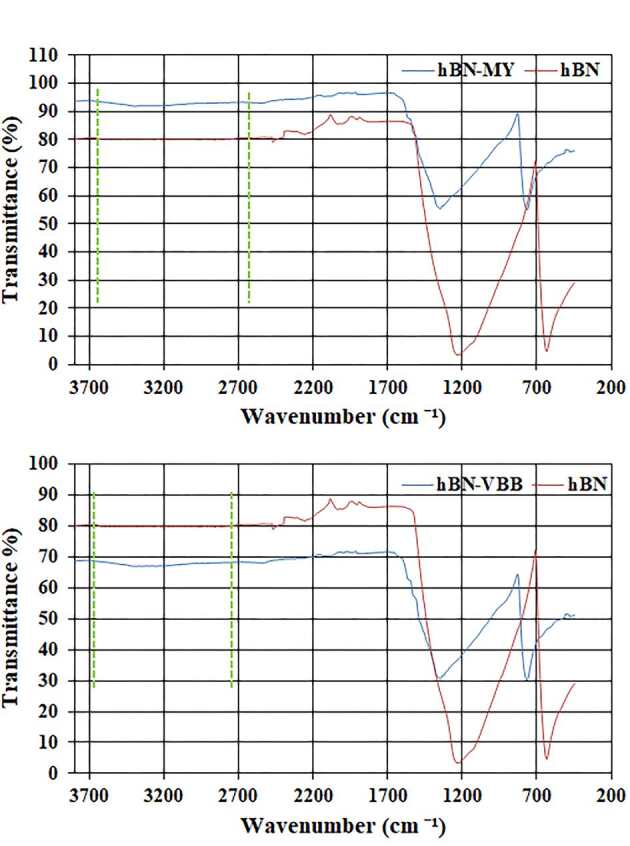
Comparative FTIR spectra of the hBN nanostructure and hBN-dye conjugate (a) for the adsorbed MY and (b) VBB dyes.

**Figure 3:**
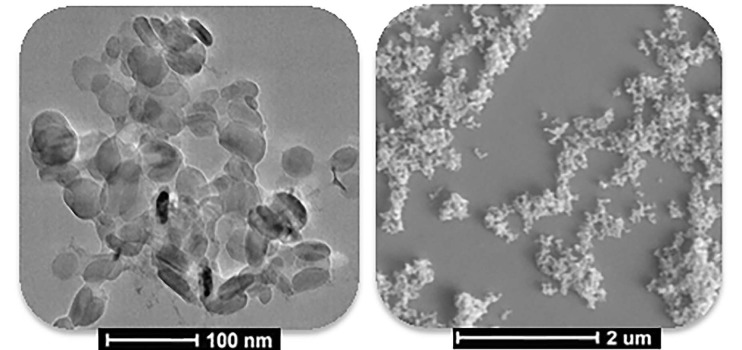
TEM (a) and SEM (b) images of the pristine hBN nanostructure.

The FTIR spectra of the hBN and hBN-dye conjugates are given in Figures 2a (for the adsorbed MY dye) and 2b (for the adsorbed VBB dye), where it can be seen that the characteristic peaks of the organic dyes were not observed on the spectra of the hBN-dye conjugates because the hBNs had strong and wide characteristic peaks. However, a slight tilt was observed in the range of 3700 and 2700 cm ^-1^ in the spectrum of the hBN-dye conjugates due to the functional groups of the adsorbed dyes. In addition, the peak intensities of the spectra of the hBN-dye conjugates significantly decreased when compared with the pristine hBN nanostructure after dye adsorption. Moreover, when studies related to the adsorbed dye onto hBN were examined in the literature, they were reported only for the FTIR spectrum of hBN [27,37–39]. Probably, the characteristic peaks of the adsorbed dye could not be observed on the spectra due to the strong and wide characteristic peaks of hBN. The visible characterization of the pristine hBN nanostructure was performed using TEM and SEM images, as shown in Figures 3a and 3b, respectively. The particle size of the pristine hBN nanostructure was around 50 nm, as shown in Figure 3a at a scale of 100 nm.

### 3.2. Mechanism of adsorption

**Figure 4:**
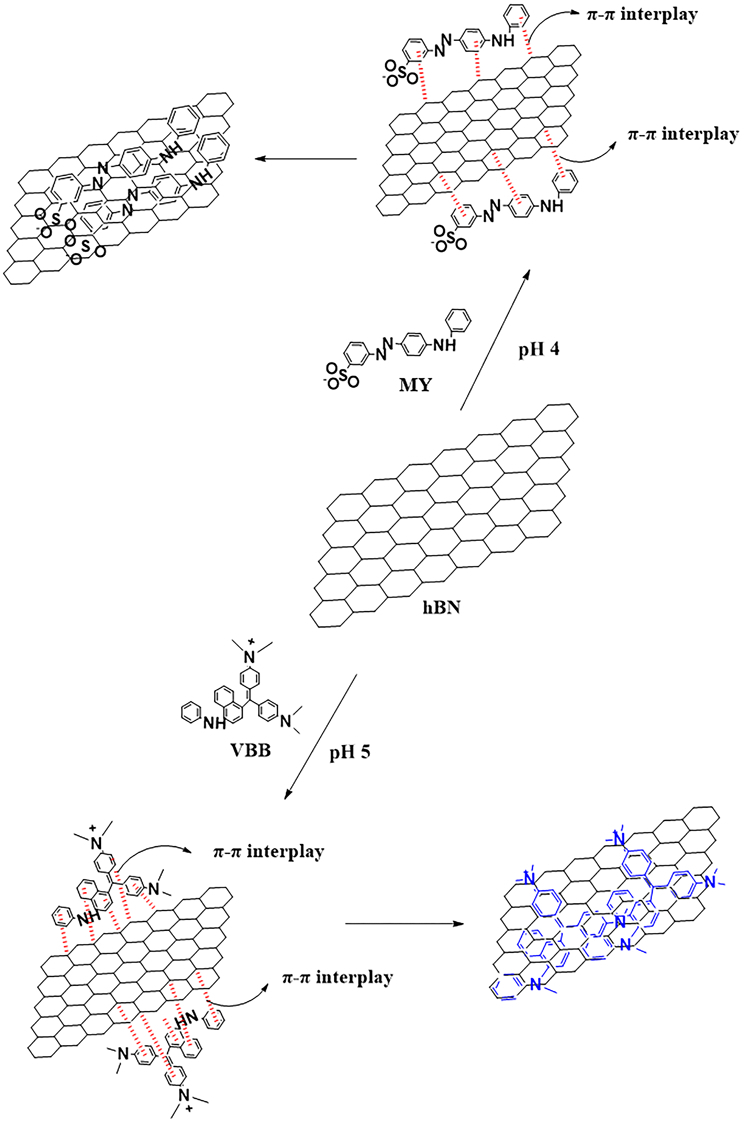
Representation of the adsorption mechanism of the adsorbed MY and VBB onto the hBN nanostructure via π-π stacking interplay.

Various factors, such as the structures of the dye and adsorbent, charges in the dye molecule, and adsorbent material effect the removal of dye molecules onto the adsorbent [39,40]. Due to a negative charge on the surface of the hBN nanostructure, it exhibited a better adsorbent property towards the cationic dye molecules than towards the anionic dye molecules. The hBN nanostructure and hBN-dye conjugates were dispersed in ultra-pure water and the surface charges were determined using zeta potential measurements before and after adsorption. The measurements of the zeta potential are shown in Table 1 and were confirmed with the described values for kinds of BN materials in the literature due to the B-OH and N-OH generated on the hBN in water [41–43]. According to Table 1, the zeta potential of the hBN decreased after the adsorption of VBB, whereas it increased after the adsorption of MY. The difference in the zeta potential change meant that the hBN nanostructure had a good adsorbent property against cationic dye. It can be estimated that VBB was better adsorbed because it is a cationic dye. Moreover, the aromatic backbone strengthened the connection between the adsorbent (hBN) and adsorbates (MY and VBB dye molecules) via π-π stacking interplay and thus, the adsorption increased and accelerated (see Figure 4) [39,40].

**Table 1:** Zeta potential measurements of the hBN nanostructure before and after adsorption of the dyes.

Sample	Zeta potential (mV)
hBN	–24.3 ± 2.05
hBN-VBB	–12.6 ± 3.13
hBN-MY	–32.9 ± 3.22

### 3.3. Adsorption studies

**Figure 5:**
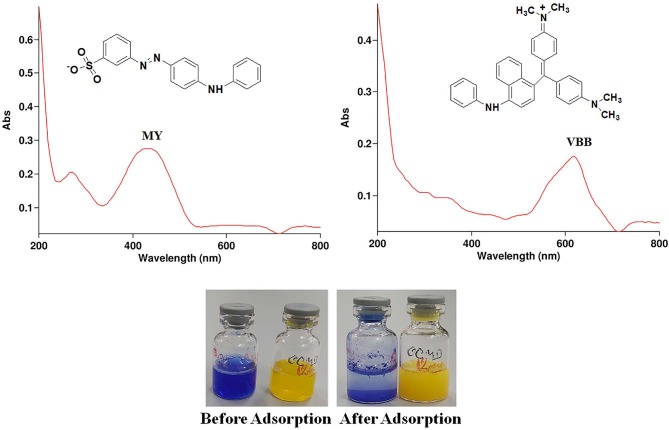
UV-Vis absorption spectra and molecular structure of MY and VBB (a), respectively, and an image of the before and after adsorption of MY and VBB onto the hBN nanostructure for 24 h (b).

The UV-Vis absorption spectra and molecular structure of the MY and VBB dyes are shown in Figure 5a. The absorbance of the supernatant solution was measured using a UV/Vis spectrophotometer at 434 and 618 nm for the MY and VBB dyes, respectively. When the adsorption of the MY and VBB dyes on the surface of the hBN nanostructure was examined, the results clearly showed that the cationic dye (VBB) adsorbed much better than the anionic dye (MY), as shown in Figure 5b. Moreover, the effects of several parameters, such as dye concentration, adsorbent dose, and effect of pH, were discussed in detail.

#### 3.3.1. Effect of pH

**Figure 6:**
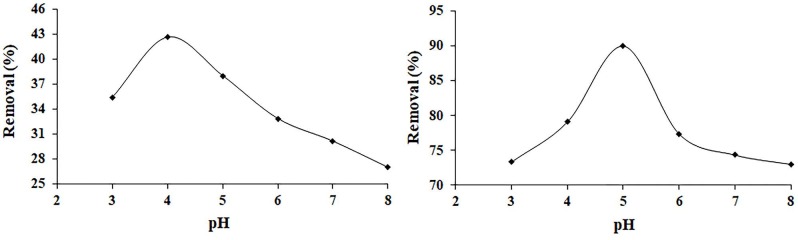
Effect of pH on the adsorption of MY (a) and VBB (b) onto the hBN nanostructure.

Adsorption studies are highly dependent on the pH value of the solution because it directly influences the surface charge of the adsorbent and the structure of the dye molecule. In other words, the pH value of the solution affects the interaction between the dye molecule and adsorbent [12,44]. Therefore, adsorption of the dye molecules (MY and VBB) onto the hBN nanostructure were investigated with apH range of 3–8. The effect of pH on the adsorption of MY was conducted with an initial dye concentration of 7 mgL ^-1^ , with 10 mg of the hBN nanostructure and at a stirring rate of 200 rpm at 25 °C for 24 h. On the other hand, the effect of pH on the adsorption of VBB was performed with an initial dye concentration of 12 mgL ^-1^ , with 10 mg of the hBN nanostructure, at 200 rpm and 25 °C for 24 h, and the results are shown in Figure 6, where it can be seen that the optimum pH value for the removal of the MY dye was determined as pH 4 and the % removal of the MY dye was calculated as 42.6% for 24 h. The optimum pH value for the removal of the VBB dye was ascertained as pH 5 and the % removal of the VBB dye was calculated as 90% for 24 h (Figure 6b).

#### 3.3.2. Effect of the dose

**Figure 7:**
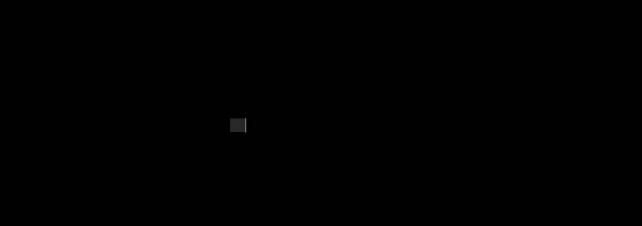
Effect of adsorbent dose on the adsorption of MY (a) and VBB (b).

The effect of the adsorbent dose was performed in the range of 5–20 mg of adsorbent. The dose experiments were performed with the initial dye concentrations onto the hBN nanostructure and at a stirring rate of 200 rpm at 25 °C for 24 h. Removal of dyes increased with an increasing adsorbent dose, as can be seen in Figure 7. Therein, 20-mg adsorbent dose removed 48.5% of the MY and 93.6% of the VBB dyes for 24 h, as shown in Figures 7a and 7b, respectively. However, the 10-mg adsorbent dose was determined as the optimum dose because it was more economical than the 20-mg adsorbent dose, and there was no significant difference between the 2 adsorbent doses.

#### 3.3.3. Effect of the initial concentration

**Figure 8:**
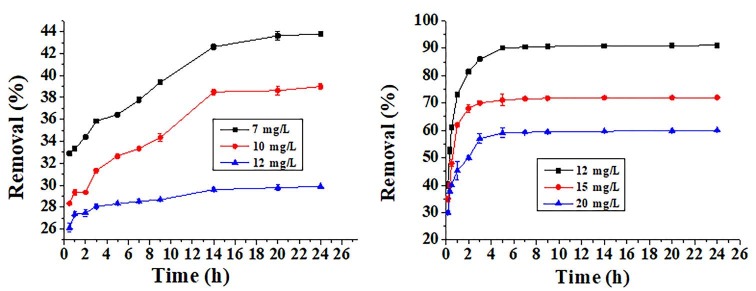
Effect of the initial dye concentration on the adsorption of MY (a) and VBB (b) dependent on time.

The effect of the initial dye concentration of MY was examined in the range of 7–12 mgL ^-1^ onto 10 mg of the hBN nanostructure for 24 h. The extent of adsorption increased over time and reached equilibrium in 14 h. In the equilibrium, the maximum % removal of the MY was calculated 42.6%, as shown in Figure 8a. Moreover, the effect of the initial dye concentration of the VBB was performed in the range of 12–20 mgL ^-1^ onto 10 mg of the hBN nanostructure for 24 h. Equilibrium was reached in 5 h and in the equilibrium, the maximum % removal of the VBB was calculated as 90% (Figure 8b). As shown in Figure 8, the VBB dye was adsorbed both faster and to a greater extent than the MY dye onto the hBN nanostructure.

### 3.4. Adsorption isotherms

Generally, the Freundlich and Langmuir equations are used for defining the adsorption isotherm model between the adsorbate and surface of an adsorbent. It was proposed to follow the Freundlich isotherm model [18], as in Eq. (3):

(3)logqe=logKf+1nlogCe

where *K*_*f*_ is the Freundlich isotherm constant (Lg ^-1^ ), *n* is the adsorption intensity or *1/n* is the heterogeneity factor, *q*_*e*_ is the amount of adsorbed dye per gram of the adsorbent at equilibrium (mgg ^-1^ ), and *C*_*e*_ is the equilibrium concentration of adsorbate (mgL ^-1^ ).

The plot of *logq* *e* versus *logC* *e* is linear. In the linear equation, a slope indicates *1/n* and an intercept indicates *logK* *f* . The results of the Freundlich isotherm model were shown in Table 2. On the other hand, if *1/n* = 1, the adsorption is linear. If the value of *n* is between 1 and 10, the adsorption process indicates favorable adsorption [45]. Moreover, if the value of *1/n* is above 1, the adsorption process indicates cooperative adsorption [46]. In this study, while the n value showed suitable adsorption for the VBB dye, it showed cooperative adsorption for the MY dye (see Table 2).

It was proposed to follow the Langmuir isotherm model [19], as in Eqs. (4) and (5):

(4)Ce=1KLqm+Ceqm(5)qm=KLb

where *q*_*m*_ is the maximum monolayer adsorption capacity of the adsorbent (mgg ^-1^ ), *K*_*L*_ is the Langmuir adsorption constant (Lmg ^-1^ ), *q*_*e*_ is the amount of adsorbed dye (mgg ^-1^ ), *C*_*e*_ is the equilibrium concentration of dye solution (mgL ^-1^ ), and the constant *b* is related to the energy or the net enthalpy of the sorption process (Lmg ^-1^ ) [18]. The Langmuir isotherm model is effective for monolayer adsorption onto the surface of adsorbents for containing a limited number of identical sites. All of the calculation results related to the Langmuir isotherm model was shown in Table 2 for both dyes.

**Table:** Langmuir and Freundlich isotherm parameters for adsorption of the MY and VBB onto the hBNnanostructure.

Dye	Langmuir constants						Freundlich constants				
	K˪(Lg ^-1^ )	b (Lmg ^-1^ )	qm(mgg ^-1^ )	R²	RMSE	∆q (%)	KF(Lg ^-1^ )	n	R²	RMSE	∆q (%)
MY	0.091	0.066	1.38	0.94	4.77	0.10	9.7E 22	0.04	0.98	1.5	0.04
VBB	0.459	0.002	250	0.95	38.2	0.07	1352.7	1.49	0.91	39.1	0.08

The correlation coefficient values ( *R* ^*2*^ ) were calculated using the Langmuir and Freundlich isotherm models for adsorption of the MY and VBB dyes. In addition to the correlation coefficient value ( *R* ^*2*^ ), the best fit isotherm model was confirmed using the residual root means quare error (RMSE) and normalized standard deviation (∆q (%))[47,48], as shown inEqs. (6) and (7):

(6)RMSE=1n-1∑n=1n(qe,exp-qe,cal)2(7)Δq(%)=∑i=1n((qe,exp-qe,cal)/qe,exp)2n-1

where *q*_*e.exp*_ (mgg−1) is the experimental adsorption capacity in the equilibrium and n is the number of data points. *q*_*e.cal*_ (mgg−1) is the calculated equilibrium adsorption capacity from the model.

Smaller values of the RMSE and ∆q (%) correspond to better curve fitting (see Table 2). According to Table 2, while the Freundlich isotherm was fitted to the isotherm model for the removal of the MY dye, the Langmuir isotherm was better when compared to the Freundlich isotherm for the removal of the VBB dye. The best fit isotherm model for adsorption of the dye was determined by considering higher R2 values, and lower RMSE and ∆q (%) values.

The dimensional constant, which is known as equilibrium parameter or separation factor, the necessary characteristics of the Langmuir equation, RL, [18], can be defined as in Eq. (8):

(8)RL=1C0.b+1

where *C*_*0*_ is the initial dye concentration (mgL ^-1^ ) and *b* is the Langmuir equilibrium constant (Lmg ^-1^ ). The value of *R*_*L*_ demonstrations the type of Langmuir isotherm, such as irreversible ( *R*_*L*_ = 0), linear ( *R*_*L*_ = 1), unfavorable ( *R*_*L*_ > 1), or favorable (0 < *R*_*L*_ < 1) [18,49]. The *R*_*L*_ values were in the range of 0 < RL < 1 for the adsorption studies of the MY and VBB dyes with different initial concentrations, indicating that the adsorption process was favorable onto the hBN nanostructure for the VBB and MY dyes.

### 3.5. Kinetic studies

In this study, 2 kinetic models were examined to investigate the mechanism of adsorption and observe the behavior of the adsorbent. The pseudo-first-order and pseudo-second-order kinetic models were tested for analyzing the experimental data. The results of the calculations related to the kinetic models are shown in Table 3. Moreover, the residual RMSE and ∆q (%) were calculated for the kinetic studies.

The pseudo-first-order was expressed by Lagergren [18,50], as in Eq. (9):

(9)ln(qe-qt)=lnqe-k1t

where *q*_*e*_ and *q*_*t*_ are amounts of dye onto the hBN nanostructure at the equilibrium and *t* time (mgg ^-1^ ), respectively, and *k*_*1*_ is the rate constant of the pseudo-first-order adsorption process (h–1). The plots of *ln(q*_*e*_ *-q*_*t*_ *)* versus *t* were used to obtain the rate constant, *k*_*1*_ , and correlation coefficient.

The pseudo-second-order was expressed by Ho and McKay [18,51], as in Eq.(10):

(10)tqt=1k2qe2+tqe

where *k*_*2*_ is the rate constant of the pseudo-second-order adsorption process (gmg ^-1^ h–1). The straight-line plots of *t/q*_*t*_ versus *t* were used to define the rate constant, *k*_*2*_ , and correlation coefficient and an intercept of the linear chart gave *1/k*_*2*_ *q*_*e*_^*2*^ . When the correlation coefficients and the values of RMSE and ∆q (%) in Table 3 were examined, the first-order kinetic model was found to be suitable for the adsorption kinetic of the MY dye. Moreover, the second-order kinetic model was found to fit better than the first-order kinetic model for the adsorption kinetic of the VBB dye. Furthermore, it was determined in the literature that the MY dye better fit the first-order [52] and the VBB dye fit well the second-order [12].

**Table 3:** Pseudo-first-order and pseudo-second-order adsorption rate constants, correlation coefficients, RMSE, ∆q (%), calculated q(e,cal), and experimental q(e,exp) values for different beginning dye concentrations of the MY and VBB dyes.

Dye	Dye	First-order kinetic model					Second-order kinetic mode				
	Initial concentration (mgL ^-1^ )	k _1_ (h ^-1^ )	qe(mgg ^-1^ )	R2	RMSE	∆q (%)	k _2_ (gmg ^-1^ h–1)	q _e,exp_ . (mgg ^-1^ )	R _2_	RMSE	∆q (%)
VBB	12	0.1017	244.8	0.197	135.9	0.319	2.20E-03	666.7	0.99	76.9	0.20
	15	0.1328	111.3	0.432	122.8	0.319	1.10E-03	833.3	0.99	90.3	0.24
	20	0.0053	10683	0.375	604.2	1.478	6.00E-04	1111.1	0.97	127	0.25
MY	7	0.1106	31.10	0.97	6.83	0.54	5.30E-03	44.8	0.93	62.4	3.13
	10	0.1159	35.47	0.89	9.25	0.24	9.20E-03	49.29	0.95	9.18	0.29
	12	0.1482	24.85	0.99	8.77	0.26	6.40E-03	54.05	0.95	9.02	0.30

## 4. Conclusion

In summary, a hBN nanostructure was successfully synthesized using boric acid. The environmentally-friendly and nontoxic material, hBN, was used for comparison of the removal of anionic and cationic dyes in an aqueous solution. This nanostructure showed poor adsorbent property for the removal of the anionic MY dye. However, it exhibited an excellent ultrafast adsorbent property for the removal of the cationic VBB dye [40]. This difference in the adsorption capacity of the anionic and cationic dyes onto the hBN nanostructure could be ascribed to the interdependent effect of electrostatic attractions. (see Table 1, the results of the zeta potential measurement). In addition, noncovalent interactions, such as π–π stacking, interplay between the adsorbent (hBN nanostructure) and adsorbate (dye molecules) made an important contribution to its adsorption capacity. Dyes contain benzene molecules that are similar to B-N rings on the plane of h-BN. Therefore, π–π stacking occurs between the benzene molecule of the dyes and B-N rings beneﬁt in the enhancement of interaction, consequently resulting in the improvement of the adsorption of the dyes (see Figure 4) [53].The % removal of the MY dye in comparison to the VBB dye was calculated as 42.6% and 90% for 10 mg of the hBN nanostructure in the case of the equilibrium, respectively. Moreover, the adsorption capacity was determined as 895.2 and 211 mgg ^-1^ for the cationic (VBB) and anionic (MY) dyes for 10 mg of the hBN nanostructure in case of the equilibrium, respectively [40]. Therefore, the high adsorption capacity and ultrafast adsorption property of the hBN towards cationic dyes make it a potentially attractive adsorbent in wastewater cleaning.
